# Glasgow Prognostic Score as a Marker of Mortality after TAVI

**DOI:** 10.21470/1678-9741-2020-0269

**Published:** 2021

**Authors:** Ozge Ozcan Abacioglu, Nermin Yildiz Koyunsever, Salih Kilic, Arafat Yildirim, Ibrahim Halil Kurt

**Affiliations:** 1 Department of Cardiology, Adana Numune Training and Research Hospital, Adana, Turkey.

**Keywords:** Transcatheter Aortic Valve Replacement, Aortic Valve Stenosis, C-Reactive Protein, Hospital Mortality, Prognosis, Serum Albumin

## Abstract

**Introduction:**

The Glasgow prognostic score (GPS) reflects host systemic inflammatory response and has been reported to be significant as a prognostic indicator in cancer-bearing patients. The aim of this study was to evaluate the predictive value of GPS in outcomes of patients with severe aortic stenosis who were treated with transcatheter aortic valve implantation (TAVI).

**Methods:**

The study population consisted of 79 patients who underwent TAVI due to severe aortic stenosis between January 2018 and March 2019 in our clinic. Echocardiographic and laboratory data were recorded before the procedure and GPS was scored as 0, 1, or 2, based on serum albumin and C-reactive protein levels. European System for Cardiac Operative Risk Evaluation II scoring system was used for risk stratification. The primary endpoints of the study were postoperative in-hospital mortality, hospitalization due to cardiac causes, or mortality within a year.

**Results:**

The 79 patients were classified into two groups according to outcomes. Fifteen patients (19%) reached the primary endpoints at one year of follow-up. Compared to the patients who did not reach the endpoints, these 15 patients were not different in terms of age, preoperative mean gradient, and ejection fraction (*P*>0.05 for all). GPS was the only laboratory parameter with statistically significant difference between the groups (*P*=0.008) and multivariate analysis showed that GPS was independent predictor of primary endpoints (*P*=0.012, odds ratio 4.51, 95% confidence interval 1.39-14.60).

**Conclusion:**

GPS is an easy, noninvasive laboratory test which may be used as a predictive biomarker for outcomes in patients undergoing TAVI.

**Table:** 

Abbreviations, acronyms & symbols				
**ALT** **AST** **AVR** **CAD** **CI** **CRP** **DM** **EF** **EuroSCORE**	**= Alanine transaminase** **= Aspartate transaminase** **= Aortic valve replacement** **= Coronary artery disease** **= Confidence interval** **= C-reactive protein** **= Diabetes mellitus** **= Ejection fraction** **= European System for Cardiac Operative Risk Evaluation**		**GFR** **GPS** **Hb** **HPL** **HT** **LDL** **OR** **pro-BNP** **TAVI**	**= Glomerular filtration rate** **= Glasgow prognostic score** **= Hemoglobin** **= Hyperlipidemia** **= Hypertension** **= Low-density lipoprotein** **= Odds ratio** **= Pro-brain type natriuretic peptide** **= Transcatheter aortic valve implantation**

## INTRODUCTION

Aortic stenosis is one of the most common valve diseases in the worldwide population ^[1]^. Although it may be clinically asymptomatic for many years, prognosis is poor when the symptoms appear ^[2]^. In symptomatic patients, aortic valve replacement (AVR) is the curative therapy because until now there has not been any medical therapy to stop the progression of the disease. Transcatheter aortic valve implantation (TAVI) is a new treatment option that is primarily applied in high-risk patients and is suggested to be applied for moderate and low risk afterwards ^[3]^. Lu et al. ^[4]^ demonstrated a significant improvement in the valve functions and New York Heart Association classification after TAVI procedure in a small group of patients over 75 years old. In another study by Kocaaslan et al., the increase in quality of life parameters measured with the 36-Item Short Form Survey, or SF-36, in the TAVI group at the end of the 3^rd^ postoperative month was greater than that in the AVR group ^[5]^.

Immunohistochemical analyses on patients with different degrees of aortic stenosis have revealed many similar points between early stage of aortic stenosis and atherosclerosis ^[6]^. The most common are inflammatory cell infiltration, high levels of lipoproteins, and calcium storage. Recent studies have shown that aortic stenosis’ development is initiated by endothelial dysfunction, inflammatory process, and lipid infiltration, whereas progression is induced by mechanical stress and genetic factors, as well as interaction between inflammatory and calcinosis processes ^[7-9]^.

The inflammation-based prognostic score termed Glasgow prognostic score (GPS), composed of serum elevation of C-reactive protein (CRP) and decrease in albumin concentration, is the most validated inflammatory risk score in cancer and predicts survival in heart failure with reduced ejection fraction, mortality in acute coronary syndrome patients, and outcomes in patients with heart failure with preserved ejection fraction ^[10,11]^. GPS was defined based on the presence of hypoalbuminemia (< 35 g/L) and elevated CRP (> 10 mg/L): if both were abnormal, the score was 2; if either was abnormal, the score was 1; if neither was abnormal, the score was 0 ^[12]^. Gassa et al. ^[13]^ found that lower serum albumin levels are correlated with both longer intensive care unit and in-hospital length of stay and 30-day mortality after TAVI in a group of 457 patients. Galante et al. determined higher CRP levels in patients with degenerative aortic stenosis requiring surgical repair ^[14]^. However, the prognostic value of GPS in patients undergoing TAVI has not been investigated. In the present study, we evaluated the significance of GPS in patients treated with TAVI.

## METHODS

### Study Population, Collection of Blood Samples, and Laboratory Measurement

One hundred and six patients with severe aortic stenosis that had been treated with TAVI in our clinic between January 2018 and March 2019 were included in this retrospective study. Twenty-seven patients who could not be reached or whose albumin or CRP levels were not found before the operation were excluded from the study. The demographic and medical characteristics of the patients were obtained from their files and in the hospital digital recording system. Routine laboratory parameters of the patients were examined before TAVI procedure and were recorded in the hospital digital system. Echocardiographies were performed using commercially available ultrasonographic equipment according to recommendations of the American Society of Echocardiography before TAVI procedure. Left ventricular ejection fraction was measured and recorded using the modified biplane Simpson method. Coronary angiography was performed before TAVI procedure in all patients. In coronary angiography, at least one of the epicardial coronary arteries had a stenosis of 50% and above and it was defined as the presence of coronary artery disease. Hypertension was defined as a systolic blood pressure ≥ 140 mmHg, diastolic blood pressure ≥ 90 mmHg, or current use of antihypertensive medication. Diabetes mellitus was defined as fasting serum glucose ≥ 126 mg/dL, hemoglobin A1c ≥ 6.5, or the use of blood glucose lowering agents. Hyperlipidemia was defined as total cholesterol value > 200 mg/dL or taking antilipidemic drugs. Patients with known inflammatory disease, autoimmune disease, or chronic liver disease, or whose CRP and albumin values were not recorded before or within the last three weeks, were excluded. According to the European System for Cardiac Operative Risk Evaluation II (EuroSCORE II) scoring system, each patient was classified as: 0-2, low-risk group; 3-5, moderate-risk group; ≥ 6, high-risk group ^[15]^.

Twelve-month follow-up data of the patients were obtained from hospital records and telephone interviews. The data of 45 patients were recorded during their check-ups, while the remaining 34 were reached by phone. Study protocol was approved by the local clinical research ethics committee and complies with the Declaration of Helsinki.

Blood samples were collected, and the laboratory measurements of serum values of CRP and albumin were performed before the operation. All patients were categorized into three groups based on the GPS score as follows: GPS 2, elevated CRP (> 10 mg/ L) and hypoalbuminemia (< 3.5 g/L); GPS 1, elevated CPR or hypoalbuminemia; GPS 0, neither elevated CPR nor hypoalbuminemia.

### Study Endpoint

Postoperative in-hospital mortality, hospitalization due to cardiac causes, or mortality within a year were defined as the primary study endpoints.

### Statistical Analysis

Levene’s test was used to determine whether variables were homogeneously distributed. Continuous variables were expressed as mean ± standard deviation and compared using analysis of variance and Kruskal-Wallis test for variables without normal distribution. Categorical variables were presented as total number and percentages and compared using the chi-square test and correlations between variables with Pearson’s correlation. Multivariate analysis using logistic regression models tested variables with *P*≤0.2 in univariate analysis and the odds ratio (OR) indicates the relative risk of the groups’ primary endpoints. A two-tailed *P*-value of < 0.05 was considered as statistically significant and 95% confidence interval (95% CI) was presented for all OR. All statistical analyses were performed using SPSS Inc. Released 2007, SPSS for Windows, Version 16.0, Chicago, SPSS Inc.

## RESULTS

A total of 79 patients (mean age, 79.5±7.8 years; 29 men [36.7%]) were included in the study. The study population had moderate-high risk for cardiac surgery with a mean EuroSCORE II of 5.85±4.44. Of these patients, two (2.5%) died during hospitalization and 13 (16.5%) died or were re-hospitalized due to cardiac causes during the follow-up period. Twenty-eight patients (35.4%) had a GPS of 0, 40 patients (50.6%) had a GPS of 1, and 11 patients (13.9%) had a GPS of 2 on admission. Table 1 shows the patients’ baseline demographic, clinical, and laboratory characteristics according to GPS.

**Table 1:** Association of GPS with patients' characteristics.

	GPS=0 (n=28)	GPS=1 (n=40)	GPS=2 (n=11)	*P* -value
Age (years)	76.3±8.9	81.5±7.1	80.7±4.7	0.026*
Male (n, %)	8 (28.5)	14 (35)	7 (63.6)	0.118
Comorbidity (n, %)				
HT	3 (10.7%)	11 (27.5%)	2 (18.1 %)	0.212
DM	3 (10.7%)	7 (17.5%)	3 (27.2%)	0.438
CAD	15 (53.5%)	18 (45%)	8 (72.7%)	0.294
HPL	10 (36)	16 (40)	4 (36)	0.574
Laboratory findings				
Hb (g/dL)	11.8±1.8	11.4±1.6	10.7±0.9	0.158
Glucose (mg/dL)	128.4±67.6	121.3±43.7	129.5±36.2	0.828
Creatinine (mg/dL)	1.0±1.2	1.0±0.5	1.0±0.2	0.971
Urea (mg/dL)	45.6±27.5	53.0±25.2	62.3±26.7	0.191
ALT (U/L)	15.6±7.8	14.4±5.6	14.9±5.9	0.764
AST(U/L)	24.2±8.2	25.0±8.2	21.3±11.8	0.475
LDL (mg/dL)	122.8±35.4	119.4±39.7	107.3±19.7	0.515
pro-BNP (pg/mL)	3793.9±4734.3	6353.3±10240.0	5011.1±6869.2	0.565
GFR (ml/m)	78.7±18.0	66.6±23.8	52.5±13.4	0.024*
Echocardiography				
EF (%)	51.4±9.9	52.0±8.8	55.5±9.1	0.515
Preoperative mean gradient (mmHg)	49.6±13.2	49.2±8.4	50.7±12.0	0.935
EuroSCORE II	4.89±2.75	4.66±2.87	7.18±5.75	0.500

ALT=alanine transaminase; AST=aspartate transaminase; CAD=coronary artery disease; DM=diabetes mellitus; EF=ejection fraction; EuroSCORE=European System for Cardiac Operative Risk Evaluation; GFR=glomerular filtration rate; GPS=Glasgow prognostic score; Hb=hemoglobin; HPL=hyperlipidemia; HT=hypertension; LDL=low-density lipoprotein; pro-BNP=pro-brain type natriuretic peptide

* Statistically significant

**Fig. 1:**
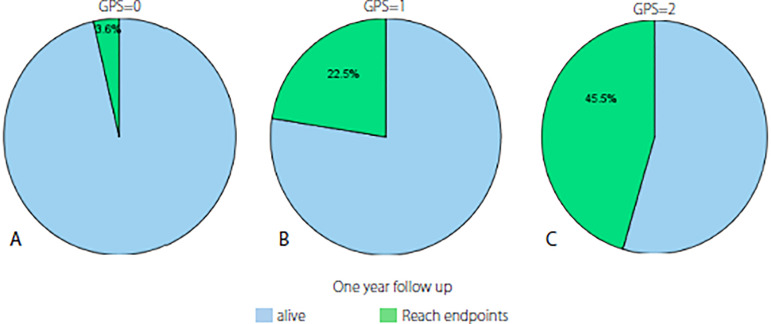
Percent of cases which reached endpoints according to the Glasgow prognostic score (GPS). A) GPS 0; B) GPS 1; C) GPS 2.

The patients who reached the endpoints (n=15) and who did not reach them were compared. The number of males and GPS values were higher in the ones who reached the endpoints (nine males [31%], six females [12%]; *P*=0.038 and *P*=0.008, respectively) and there was a moderate correlation between GPS and the endpoints (r=0.349, *P*=0.002) Percent of cases who reached end-points according to GPS were shown in Figure 1. Other baseline, clinical, and laboratory characteristics did not differ significantly between the groups (Table 2).

**Table 2:** Characteristics and echocardiographic and laboratory findings of patients according to primary endpoints.

	Alive (n= 64)	Reach endpoints (n=15)	*P* -value
Age, years	79.5±8.2	79.8±6.3	0.130
Gender (male, %)	20 (69)	9 (31)	0.038
HT (n, %)	13 (20.3)	3 (20)	0.956
DM (n, %)	9 (14)	4 (26.6)	0.248
CAD (n, %)	33 (51.5)	8 (53.3)	0.747
EF (%)	52.2±9.2	52.6±9.0	0.431
Preoperative gradient (mmHg)	49.4±10.8	50.1±9.9	0.095
Albumin (mg/dL)	36.3±3.2	33.0±4.8	0.219
CRP (mg/L)	8.2±12.8	8.0±7.1	0.396
Urea (mg/dL)	53.0±28.2	46.0±16.8	0.492
Creatinine (mg/dL)	0.8±0.5	0.9±0.3	0.472
Hb (g/dL)	11.5±1.7	10.9±1.0	0.973
Glucose (mg/dL)	125.0±53.3	124.6±46.3	0.332
AST (U/L)	24.1±8.4	24.7±10.3	0.297
ALT (U/L)	15.1±6.9	14.1±4.0	0.565
LDL (mg/ dL)	118.3±35.5	122.3±39.7	0.392
Pro-BNP (pg/mL)	4572.0±7213.5	8648.5±11829.3	0.435
GFR (ml/m)	68.7±22.0	63.8±24.0	0.368

ALT=alanine transaminase; AST=aspartate transaminase; CAD=coronary artery disease; CRP=C-reactive protein; DM=diabetes mellitus; EF=ejection fraction; GFR=glomerular filtration rate; Hb=hemoglobin; HT=hypertension; LDL=low-density lipoprotein; pro-BNP=pro-brain type natriuretic peptide

In an analysis of the prognostic factors for endpoints, age, gender, preoperative mean gradient, ejection fraction, pro-brain type natriuretic peptide (pro-BNP), and GPS were analysed (Table 3). Univariate analysis revealed that age (*P*=0.140) and GPS (*P*=0.056) were prognostic factors for primary endpoints. In the multivariate analysis, GPS (OR 4.383, 95% CI 1.636-11.739; *P*=0.003) was the only prognostic factor for endpoints.

**Table 3:** Univariate and multivariate analysis of prognostic factors associated with primary endpoints in patients who underwent TAVI.

	Univariate analysis			Multivariate analysis		
	OR	95% CI	*P* -value	OR	95% CI	*P* -value
Age	0.882	0.747-1.042	0.140*	0.976	0.898-1.060	0.558
Gender	0.897	0.060-13.388	0.937	-	-	-
EF	0.918	0.770-1.095	0.341	-	-	-
Preoperative gradient	0.975	0.841-1.131	0.741	-	-	-
pro-BNP	1.000	1.000-1.000	0.584	-	-	-
GPS	11.789	0.940-147.837	0.056*	4.383	1.636-11.739	0.003*

CI=confidence interval; EF=ejection fraction; GPS=Glasgow prognostic score; OR=odds ratio; pro-BNP=pro-brain type natriuretic peptide; TAVI=transcatheter aortic valve implantation

* Statistically significant

## DISCUSSION

In the present study, the in-hospital mortality rate of TAVI was 2.5% and the re-hospitalization or non-hospital mortality during the one-year follow-up period was 16.5%. Patients with higher GPS values had higher rates of primary endpoints.

Calcific aortic stenosis is known to be a chronic inflammatory disease and there are studies supporting that endothelial injury or dysfunction are early signs of the disease. Aortic valve interstitial cells have been shown to play an important role in the pathogenesis of calcific aortic stenosis by leading to osteogenic protein expression in response to proinflammatory cytokine stimulation ^[16]^. In a study by Lee et al. ^[17]^, the interleukin-1 receptor antagonist, an important anti-inflammatory defense mechanism in the aortic valve, was found throughout non-stenotic aortic valve leaflets, and it was absent in leaflets from stenotic aortic valves. Dweck et al. ^[18]^ demonstrated that both calcification and inflammation are increased in patients with aortic stenosis compared with controls in a positron emission tomography study. Consequently, the common point of these studies is that inflammation should be taken into consideration in the pathophysiology of aortic stenosis and the progress of the disease.

There are studies showing many factors determining the prognosis of aortic stenosis such as age, ejection fraction (increased left ventricular fibrosis), gender, and serum urea level ^[19-21]^. It is assumed that the disease is in the inflammatory process; GPS, which has been used prognostically in many cancer types recently and is an inflammatory marker, may also be among these factors.

There are a few studies about GPS in cardiology. Cho et al. ^[22]^ investigated the modified GPS on survival of patients with heart failure with reduced ejection fraction and found out that increase in modified GPS was associated with adverse outcome. In another study by Bolat et al., modified GPS and N-terminal pro-BNP were independent predictors of primary endpoint in multivariate analysis in heart failure with preserved ejection fraction ^[23]^. Furthermore, high GPS values were determined in patients with acute coronary syndrome with both total and cardiovascular mortalities ^[24]^.

In our study, we showed that the high GPS values detected before the procedure in patients undergoing TAVI may be related to the increase in mortality and hospitalization frequency in and after hospital discharge. Kong et al. ^[25]^ demonstrated that myocardial fibrosis detected by cardiovascular magnetic resonance has been associated with increased all-cause mortality after AVR. In our study, there was not any difference between the patients’ ejection fraction levels, and serum urea levels were similar between the groups, but age was associated with bad prognosis in univariate analysis. However, in multivariate analysis, only GPS values predicted primary endpoints in these patients.

### Limitations

Our study had some limitations. First, it was a single-center study, and the number of participants was low. Second, the follow-up period was short. Furthermore, the ejection fraction was measured by echocardiography only; cardiac magnetic resonance or strain could be used. Also, the design of the study was retrospective, and further prospective studies are needed

## CONCLUSİON

TAVI is becoming more common and GPS value, which is a noninvasive, easy inflammatory marker, can be used in this group of patients to determine their prognosis.

**Table:** 

Authors' roles & responsibilities	
OOA	Substantial contributions to the conception or design of the work; or the acquisition, analysis, or interpretation of data for the work; drafting the work or revising it critically for important intellectual content; final approval of the version to be published
NYK	Substantial contributions to the conception or design of the work; final approval of the version to be published
SK	Drafting the work or revising it critically for important intellectual content; final approval of the version to be published
AY	Final approval of the version to be published
IHK	Final approval of the version to be published
